# A Mathematical Model to Study the Potential Hepatitis B Virus Infections and Effects of Vaccination Strategies in China

**DOI:** 10.3390/vaccines11101530

**Published:** 2023-09-27

**Authors:** Chuanqing Xu, Yu Wang, Kedeng Cheng, Xin Yang, Xiaojing Wang, Songbai Guo, Maoxing Liu, Xiaoling Liu

**Affiliations:** 1 School of Science, Beijing University of Civil Engineering and Architecture, Beijing 100044, China; 2 Mathematics Department, Hanshan Normal University, Chaozhou 521041, China

**Keywords:** HBV, potential HBV infections, stability analysis, reproduction number, vaccine

## Abstract

Motivations: Hepatitis B is a potentially life-threatening infectious disease caused by the hepatitis B virus (HBV). Approximately 390,000 people in China die from HBV-related diseases each year. Around 86 million individuals suffer from infections of the hepatitis B virus, accounting for about 6% of the total population in the region. There are approximately 30 million chronic infections. From 2002 to 2007, China’s government took part in “The Global Alliance for Vaccines and Immunization (GAVI)” initiative, which helped reduce cases of chronic HBV infections among children. However, incidences of hepatitis B remain persistently high in China. Accurately estimating the number of potential HBV infections is crucial for preventing and controlling the transmission of the hepatitis B virus. Up until now, there were no studies of potentially infectious hepatitis B virus infections. Methods: this study was based on data from the National Bureau of Statistics of China from 2003 to 2021; a dynamic model was built, which included a compartment for potentially infectious hepatitis B virus infections. The parameters in the model were fitted using a combination of nonlinear least-squares and genetic algorithm methods. Results: the calculated reproduction number for hepatitis B virus transmission within the population is $R_{c}$ = 1.741. Considering the existing vaccine inefficiency rate of 0.1, the model estimates there are 449,535 (95%CI [415,651, 483,420]) potentially infectious hepatitis B virus infections, constituting 30.49% of total hepatitis B cases. Date fitting using MATLAB reveals that increasing the rate of hepatitis B vaccinations can effectively reduce the number of infections. Conclusions: the results reveal that the number of potential infectious hepatitis B virus infections is so high that the number of hepatitis B patients persistently rises in China. To better control the transmission of the hepatitis B virus, an optional prevention and control strategy is needed to increase the vaccination of different age groups, and it is necessary to help the public correctly understand the transmission of hepatitis B and ensure adequate protection.

## 1. Introduction

**Figure 1:**
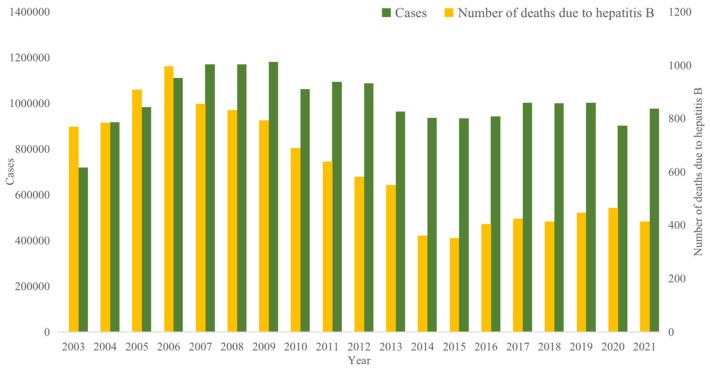
The number of hepatitis B cases and deaths in China from 2003 to 2021.

Hepatitis B (hereinafter referred to as “HBV”) is a potentially life-threatening infection of the liver caused by the hepatitis B virus [1]. According to data from the World Health Organization [2], approximately 2 billion people worldwide are infected with the hepatitis B virus, with around 250 million individuals carrying the hepatitis B virus. Each year, about 1 million people die from chronic active hepatitis, cirrhosis, or primary liver cancer associated with HBV. The World Health Organization has classified HBV infection as one of the top ten causes of global mortality. China ranks among the countries with the heaviest burden of HBV infections; data for new annual HBV cases from 2003 to 2021 [3] is shown in Figure 1 and are available on the website. On average, there are 1,007,634 new cases each year; the lowest number of new cases was recorded in 2003 at 719,011, and the highest was in 2007 at 1,169,946. Additionally, the mortality rate attributed to liver cancer resulting from HBV is the highest in China. On average, there are 614 deaths annually. The lowest number of deaths was recorded in 2015 at 352, and the highest was in 2006 at 995, as shown in Figure 1.

Hepatitis B is a viral infection that adversely affects the liver. The primary modes of transmission for the hepatitis B virus include sexual contact, mother-to-child transmission, father-to-child transmission, medical transmission, and blood transmission, among others. However, the hepatitis B virus is not spread through the following means: sharing utensils, breastfeeding, hugging, kissing, shaking hands, coughing, sneezing, or engaging in activities such as playing in public swimming pools [2].

The incubation period for the hepatitis B virus typically ranges from 1 to 6 months, with an average of 3 months. During the incubation period, individuals are contagious. Vaccination provides effective prevention against infection with the hepatitis B virus, but the vaccine’s protection might be ineffective after a period of time, potentially leading to the risk of re-infection with hepatitis B. Population mobility can exacerbate the spread of the hepatitis B virus. As long as the viral load reaches a certain concentration and the virus is shed, infections of the hepatitis B virus remain contagious at any stage.

It is impossible to clinically differentiate hepatitis B from hepatitis caused by other viruses. Therefore, a laboratory test is necessary to confirm the diagnosis. Various blood tests can be used to diagnose and monitor hepatitis B patients. Following exposure to the bodily fluids of someone with hepatitis B, observation for 6 months and a “five-item test” for hepatitis B (HBsAg, HBsAb, HBeAg, HBeAb, and HBcAb) are necessary to determine infection status [1]. Hepatitis B typically begins with subtle onset symptoms, presenting as general fatigue, weariness, loss of appetite, abdominal discomfort, and in some cases, a few patients might experience nausea and vomiting. Jaundice or mild jaundice is present, and examinations may reveal hepatomegaly. Tenderness upon palpation, splenomegaly, and occasional liver area pain are observed in a small number of patients [4]. Currently, there are two types of hepatitis B vaccines available for immunization: yeast-based vaccines and recombinant hepatitis B gene vaccines [5].

From 2002 to 2007, the World Health Organization and the Chinese Ministry of Health jointly implemented the China-GAVI project, which involved supplementary hepatitis B vaccination for adolescents in 12 western provinces of China. This initiative aimed to reduce the high incidence of hepatitis B in the country and achieved favorable outcomes. The project encompassed 12 provinces and autonomous regions in western China, including Sichuan, Guizhou, Yunnan, Tibet, Chongqing, Shaanxi, Gansu, Qinghai, Ningxia, Xinjiang, Guangxi, Inner Mongolia, as well as all counties in the 10 provinces of Hunan, Hubei, Shanxi, Jiangxi, Anhui, Henan, Heilongjiang, Hainan, Hebei and Jilin (collectively referred to as the 10 non-western provinces). The target population for the project consisted of all newborns in the aforementioned areas during the project’s implementation [6]. After 2008, there is a significant decline in the number of new hepatitis B cases, but the incidence rate remained persistently high. Beginning from 2015, the national hepatitis B case count has shown a continuous and slight increase. Given that childhood hepatitis B vaccine coverage has reached 100%, and assuming that the adults diagnosed with contagious hepatitis B would consciously manage their sexual behavior to prevent further transmission, the ongoing elevated and even slightly increasing number of hepatitis B cases may be largely attributed to potential infections spreading the virus. Therefore, accurately assessing the quantity of potentially contagious hepatitis B infections and exploring reinforced vaccination strategies for susceptible individuals holds crucial real-world significance for the prevention and control of hepatitis B.

Many researchers have, both domestically and internationally, employed mathematical models to study the transmission process of the hepatitis B virus and the impact of preventive measures on its spread. Su et al. developed a dynamic model of hepatitis B virus transmission with mechanisms of vaccination and vertical transmission [7]; Sun et al. analyzed the effect of physical examination and immunization on hepatitis B [8]; Li et al. established a mathematical model for the pharmacological blockade of HBV transmission between mothers and infants during maternal pregnancy [9]; Liu et al. considered a SIRS infectious disease model with an age structure and continuous vaccination [10]; Tao YJ et al. considered a model of hepatitis B virus transmission with vertical transmission and a period of infection when incidence transmission was nonlinear [11]; Zhao T. et al. constructed a hepatitis B kinetic model and analyzed the data of new cases of hepatitis B in Xinjiang from 2005 to 2014 [12]; O’Leary, C. et al. developed mathematical models of HBV drug treatment and immunization to compare the effects of vaccination and treatment [13]; Jianhua Pang et al. developed a model to explore the impact of vaccination and other HBV infection control measures [14]; Habenom et al. developed a fractional order mathematical model of hepatitis B virus transmission and studied possible strategies for hepatitis B vaccination and isolation control [15]; Ramses Djidjou Demasse et al. developed a model of the age structure of HBV transmission that included both symptomatic and asymptomatic infections [16]; Qiang Li et al. developed a dynamic model with seven cell types based on the biological mechanisms of viral replication and the host immune response, and this model predicted that timely long-term therapy was needed to reduce the symptoms of HBV and to maintain the benefits of treatment [17]; and Goyal, A. et al. developed novel mathematical models that incorporated these key biological processes and analyzed them both analytically and numerically, and the analysis further suggested the existence of some form of a selective advantage of infected hepatocytes containing only IDNA to explain the viral dynamics observed during antiviral treatment and the transition from peak to acute infection [18]. Since the implementation of the China-GAVI project from 2002 to 2007, the rate of chronic HBV infection in children has decreased. However, the annual incidence of new cases continues to increase in China. We believe that this is primarily due to potentially infectious hepatitis B virus infections in those who possess infectivity.

In this study, a dynamic model based on the pathogenesis of hepatitis B is developed to assess the number of potentially infectious hepatitis B virus infections. The model employs a combination of nonlinear least-squares and genetic algorithm methods to fit certain parameters, allowing for the calculation of the reproduction number of the hepatitis B virus in the population. MATLAB was used to analyze changes in the number of incidences, thereby exploring effective strategies for controlling hepatitis B virus transmission through vaccination.

This paper is divided into four sections. In the Section 1, an introduction is provided regarding the transmission of the hepatitis B virus in China; the Section 2 establishes the model, explaining the significance of each parameter and conducting model analysis; the Section 3 conducts simulation analysis, exploring the effects of the parameters on potentially infectious hepatitis B virus infections K, the proportion of K, $R_{c}$ and their impacts; the Section 4 discusses the conclusions drawn from this study. 

## 2. Methods

### 2.1. Model Building

**Figure 2:**
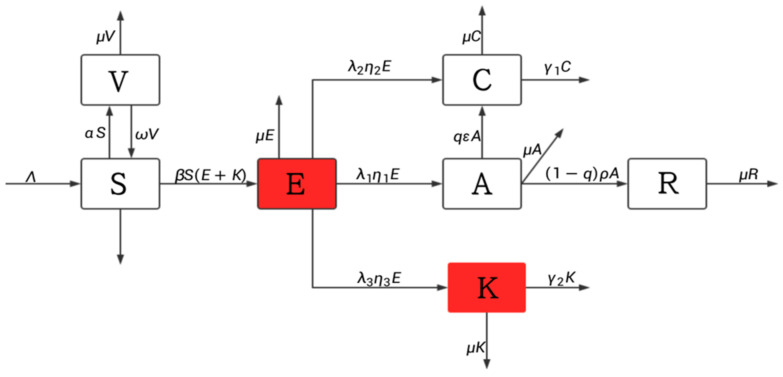
A flow diagram of hepatitis B virus transmission.

The main objective of this paper is to accurately estimate the number of potentially infectious hepatitis B virus infections and establish a dynamic model that includes potentially infectious hepatitis B virus infections based on the transmission mechanism of the hepatitis B virus. Given that the mortality rate of acute hepatitis B patients is extremely low, with more than 90% being cured [19], this study does not consider mortality due to acute hepatitis B infection. About 88% of acute hepatitis B infections transition into chronic hepatitis B infections. Since the recovery rate of chronic hepatitis B infections is very low and those who recover are prone to becoming hepatitis B patients again, this study does not account for recovery among chronic hepatitis B infections. Patients affected with both acute and chronic hepatitis B infections consciously control their sexual behavior due to their awareness of being virus carriers, so it can be assumed that they no longer possess infectivity. Hepatitis B is contagious during the incubation period, and potential infections of hepatitis B virus also exhibit infectivity. The population is divided into seven compartments: susceptible individuals S, exposed E, acute hepatitis B infections A, chronic hepatitis B infections C, potentially infectious hepatitis B virus infections K, recovered individuals R, and vaccinated individuals V. Based on the process of the hepatitis B virus, the following flow chart can be established and is shown in Figure 2:

The meaning of each parameter symbol is as follows: Λ is the number of new births rate every year; α is the vaccination rate; ω is the vaccine failure rate; β is the base transmission rate; ε is the transfer rate of A to C; ρ is the transfer rate from A to R; μ is the natural mortality rate; $\lambda_{1}$ is the conversion rate of E to A; $\lambda_{2}$ is the conversion rate from E to C; $\lambda_{3}$ is the conversion rate of E to K; $\gamma_{1}$ is the mortality rate of chronic HBV infections due to disease; $\gamma_{2}$ is the mortality rate of patients in K due to disease; $\eta_{1}$ is the transfer rate from E to A; $\eta_{2}$ is the transfer rate from E to C; $\eta_{3}$ is the transfer rate from E to K; and *q* is the proportion of people with acute infections who become chronically infected. All the parameters are nonnegative. 

Based on Figure 2, the following dynamic model of hepatitis B virus transmission was developed:

(1) $$\left\{\begin{array}{c} \;\frac{dS}{dt}=\Lambda +\omega V-\alpha S-\beta S(E+K)-\mu S,\;\;\;\;\;\;\;\;\;\;\;\;\;\;\;\;\;\;\;\;\;\;\;\;\;\;\;\;\;\;\;\;\;\;\;\;\;\;\;\;\;\;\;\;\;\;\;\;\;\;\;\;\;\;\;\;\;\;\;\;\;\;\;\;\;\;\;\;\;\;\;\;\;\;\;\;\;\;\;\;\;\;\;\;\;\;\;\;\;\\ \frac{dE}{dt}=\beta S\left(E+K\right)-\lambda_{1}\eta_{1}E-\lambda_{2}\eta_{2}E-\lambda_{3}\eta_{3}E-\mu E,\;\;\;\;\;\;\;\;\;\;\;\;\;\;\;\;\;\;\;\;\;\;\;\;\;\;\;\;\;\;\;\;\;\;\;\;\;\;\;\;\;\;\;\;\;\;\;\;\;\;\;\;\;\;\;\;\;\;\;\;\;\;\;\;\;\;\;\;\;\;\;\\ \begin{array}{c} \frac{dA}{\;dt}=\lambda_{1}\eta_{1}E-q\varepsilon A-\left(1-q\right)\rho A-\mu A,\;\;\;\;\;\;\;\;\;\;\;\;\;\;\;\;\;\;\;\;\;\;\;\;\;\;\;\;\;\;\;\;\;\;\;\;\;\;\;\;\;\;\;\;\;\;\;\;\;\;\;\;\;\;\;\;\;\;\;\;\;\;\;\;\;\;\;\;\;\;\;\;\;\;\;\;\;\;\;\;\;\;\;\;\;\;\;\;\;\;\;\\ \;\frac{dC}{dt}=\lambda_{2}\eta_{2}E+q\varepsilon A-\gamma_{1}C-\mu C,\;\;\;\;\;\;\;\;\;\;\;\;\;\;\;\;\;\;\;\;\;\;\;\;\;\;\;\;\;\;\;\;\;\;\;\;\;\;\;\;\;\;\;\;\;\;\;\;\;\;\;\;\;\;\;\;\;\;\;\;\;\;\;\;\;\;\;\;\;\;\;\;\;\;\;\;\;\;\;\;\;\;\;\;\;\;\;\;\;\;\;\;\;\;\;\;\;\;\;\;\;\;\;\\ \begin{array}{c} \frac{dK}{dt}=\lambda_{3}\eta_{3}E-\gamma_{2}K-\mu K,\;\;\;\;\;\;\;\;\;\;\;\;\;\;\;\;\;\;\;\;\;\;\;\;\;\;\;\;\;\;\;\;\;\;\;\;\;\;\;\;\;\;\;\;\;\;\\ \frac{dR}{dt}=(1-q)\rho A-\mu R,\;\;\;\;\;\;\;\;\;\;\;\;\;\;\;\;\;\;\;\;\;\;\;\;\;\;\;\;\;\;\;\;\;\;\;\;\;\;\;\;\;\;\;\;\;\;\;\;\;\;\\ \frac{dV}{dt}=\alpha S-\omega V-\mu V.\;\;\;\;\;\;\;\;\;\;\;\;\;\;\;\;\;\;\;\;\;\;\;\;\;\;\;\;\;\;\;\;\;\;\;\;\;\;\;\;\;\;\;\;\;\;\;\;\;\;\;\; \end{array}\;\;\;\;\;\;\;\;\;\;\;\;\;\;\;\;\;\;\;\;\;\;\;\;\;\;\;\;\;\;\;\;\;\;\;\;\;\;\;\;\;\;\;\;\;\;\;\;\;\;\;\;\;\;\;\;\;\;\;\;\;\;\;\;\;\;\; \end{array} \end{array}\right.$$

### 2.2. Control Reproduction Number

The control reproduction number refers to the number of secondary cases after an infected individual enters the susceptible population [20]. The next generation matrix method is used to calculate it.

The disease state of the system (1) is *E*, *A*, *C*, *K*, to calculate the number of control reproduction of system (1), let

$$F=\left[\begin{array}{c} \beta S(E+K)\\ 0\\ \begin{array}{c} 0\\ 0 \end{array} \end{array}\right],V=\left[\begin{array}{c} \lambda_{1}\eta_{1}E+\lambda_{2}\eta_{2}E+\lambda_{3}\eta_{3}E+\mu E\\ q\varepsilon A+\left(1-q\right)\rho A+\mu A-\lambda_{1}\eta_{1}E\\ \begin{array}{c} \gamma_{1}C+\mu C-\lambda_{2}\eta_{2}E-q\varepsilon A\;\\ \gamma_{2}K+\mu K-\lambda_{3}\eta_{3}E \end{array} \end{array}\right].$$

The Jacobi matrices of F, V at the disease-free equilibrium point $P_{0}$ are:

$$F=\left[\begin{array}{c} \;\beta S_{0}\;\;\;\begin{array}{ccc} 0 & 0 & \beta S_{0} \end{array}\\ 0\begin{array}{ccc} \;\;\;\;0 & 0 & 0 \end{array}\\ \begin{array}{c} 0\begin{array}{ccc} \;\;\;\;0 & 0 & 0 \end{array}\\ 0\begin{array}{ccc} \;\;\;\;0 & 0 & 0 \end{array} \end{array} \end{array}\right],$$

$$V=\left[\begin{array}{ccc} \lambda_{1}\eta_{1}+\lambda_{2}\eta_{2}+\lambda_{3}\eta_{3}+\mu & 0 & \begin{array}{cc} 0\;\;\;\;\;\;\;\;\;\;\;\;\;\;\;\;\;\;\;\;\;\;\; & 0 \end{array}\\ -\lambda_{1}\eta_{1} & q\varepsilon +\left(1-q\right)\rho +\mu & \begin{array}{cc} 0\;\;\;\; & \;\;\;\;\;\;\;\;\;\;\;\;\;\;\;\;\;\;\;0 \end{array}\\ \begin{array}{c} -\lambda_{2}\eta_{2}\\ -\lambda_{3}\eta_{3} \end{array} & \begin{array}{c} -q\varepsilon \\ 0 \end{array} & \;\;\;\;\;\;\begin{array}{c} \begin{array}{cc} \gamma_{1}+\mu & \;\;\;\;\;\;\;\;\;\;\;\;\;\;\;\;\;\;0\;\;\;\;\;\;\;\;\;\; \end{array}\\ \begin{array}{cc} \;\;0\;\;\;\;\;\;\;\; & {\;\;\;\;\;\;\;\;\;\;\;\;\gamma }_{2}+\mu \end{array} \end{array} \end{array}\right].$$

At the disease-free equilibrium point, we can derive

$$FV^{-1}=\left[\begin{array}{ccc} \frac{\beta S_{0}}{\lambda_{1}\eta_{1}+\lambda_{2}\eta_{2}+\lambda_{3}\eta_{3}+\mu }+\frac{\beta S_{0}\lambda_{3}\eta_{3}}{\left(\gamma_{2}+\mu \right)\left(\lambda_{1}\eta_{1}+\lambda_{2}\eta_{2}+\lambda_{3}\eta_{3}+\mu \right)} & 0 & 0\;-\frac{\beta S_{0}}{{\;\gamma }_{2}+\mu }\\ 0 & 0 & 0\;\;\;\;\;\;\;\;\;\;0\;\;\;\;\;\;\;\;\\ \begin{array}{c} 0\\ 0 \end{array} & \begin{array}{c} 0\\ 0 \end{array} & \begin{array}{c} 0\;\;\;\;\;\;\;\;\;\;0\;\;\;\;\;\;\;\;\\ 0\;\;\;\;\;\;\;\;\;\;0\;\;\;\;\;\;\;\; \end{array} \end{array}\right]$$

The value of the control reproduction number $R_{c}$ is the spectral radius of $FV^{-1}$, from which we can calculate

$$R_{c}=\rho \left(FV^{-1}\right)=\frac{\beta S_{0}\left(\gamma_{2}+\mu +\lambda_{3}\eta_{3}\right)}{\left(\gamma_{2}+\mu \right)\left(\lambda_{1}\eta_{1}+\lambda_{2}\eta_{2}+\lambda_{3}\eta_{3}+\mu \right)}.$$

### 2.3. Disease-Free Equilibrium Point and Its Stability

Clearly, there is a disease-free equilibrium point $P_{0}=\left(S_{0}{,0,0,0,0,0,V}_{0}\right)$ in the system (1). Where

$$S_{0}=\frac{\left(\omega +\mu \right)\Lambda }{\mu \left(\alpha +\mu +\omega \right)},V_{0}=\frac{\alpha \Lambda }{\alpha \mu +\mu^{2}+\mu \omega }.$$

**Theorem** **1.**  *The disease-free equilibrium point* $P_{0}$ *of the system (1) is locally asymptotically stable when* $R_{c}<1$*; it is unstable when*$\;R_{c}>1$.

**Proof.**  The Jacobi matrix of the system (1) at the disease-free equilibrium point $P_{0}$ is

$$\left[\begin{array}{ccc} -\alpha -\mu & -\beta S_{0} & 0\\ 0 & \beta S_{0}-\lambda_{1}\eta_{1}-\lambda_{2}\eta_{2}-\lambda_{3}\eta_{3}-\mu \; & 0\\ \begin{array}{c} 0\\ 0\\ \begin{array}{c} \begin{array}{c} 0\\ 0 \end{array}\\ \alpha \end{array} \end{array} & \begin{array}{c} \lambda_{1}\eta_{1}\\ \lambda_{2}\eta_{2}\\ \begin{array}{c} \begin{array}{c} \lambda_{3}\eta_{3}\\ 0 \end{array}\\ 0 \end{array} \end{array} & \begin{array}{c} -q\varepsilon -\left(1-q\right)\rho -\mu \\ q\varepsilon \\ \begin{array}{c} 0\\ \begin{array}{c} \left(1-q\right)\rho \\ 0 \end{array} \end{array} \end{array} \end{array}\;\;\;\;\;\begin{array}{ccc} 0 & \;\;\begin{array}{cc} -\beta S_{0} & \;\;\;0 \end{array} & \omega \\ 0 & \;\;\;\;\;\begin{array}{cc} \beta S_{0}\;\; & \;\;0 \end{array} & 0\\ \begin{array}{c} 0\\ -\gamma_{1}-\mu \\ \begin{array}{c} \begin{array}{c} 0\\ 0 \end{array}\\ 0 \end{array} \end{array} & \begin{array}{c} \;\;\;\;\;\begin{array}{cc} \;0\;\;\;\; & \;\;\;0 \end{array}\\ \;\;\;\;\;\;\begin{array}{cc} 0 & \;\;\;\;\;\;\;0 \end{array}\\ \begin{array}{c} \begin{array}{cc} -\gamma_{2}-\mu & 0 \end{array}\\ \;\;\;\;\;\;\begin{array}{c} \begin{array}{cc} 0 & \;\;\;\;-\mu \end{array}\\ \begin{array}{cc} 0 & \;\;\;\;\;\;\;\;0 \end{array} \end{array} \end{array} \end{array} & \begin{array}{c} 0\\ 0\\ \begin{array}{c} \begin{array}{c} 0\\ 0 \end{array}\\ -\omega -\mu \end{array} \end{array} \end{array}\right].$$

The characteristic polynomial of this matrix is:

$$f\left(\phi \right)=\left(\;\phi +\mu \right)\left(\phi +q\varepsilon +\left(1-q\right)\rho \;+\mu \right)\left(\phi +\gamma_{1}+\mu \right)\left[\phi^{2}+\left(\omega +2\mu +\alpha \right)\phi +\mu \left(\alpha +\omega +\;\;\;\;\mu \right)\right]\;\;\;\;\;\;\;\;\;\;\;\;[\phi^{2}+\left(-\beta S_{0}+\lambda_{1}\eta_{1}+\lambda_{2}\eta_{2}+\lambda_{3}\eta_{3}+\mu {+\gamma }_{2}+\mu \right)\phi -\beta S_{0}\gamma_{2}+\lambda_{1}\eta_{1}\gamma_{2}+\lambda_{2}\eta_{2}\gamma_{2}+{\;\;\;\;\;\lambda }_{3}\eta_{3}\gamma_{2}\;+\mu \gamma_{2}-\beta S_{0}\mu +\lambda_{1}\eta_{1}\mu +\lambda_{2}\eta_{2}\mu +\lambda_{3}\eta_{3}\;\mu +\mu^{2}-\beta S_{0}\lambda_{3}\eta_{3}]$$

Clearly

$$\phi_{1}=-\mu <0,$$

$$\phi_{2}=-\gamma_{1}-\mu <0,$$

$$\phi_{3}=-q\varepsilon -\left(1-q\right)\rho -\mu <0.$$

The other two eigenvalues $\phi_{4},\phi_{5}$ satisfy the equation

$$f_{1}\left(\phi \right)=\phi^{2}+A_{1}\phi +A_{2},$$

where

$$A_{1}=\omega +2\mu +\alpha ,\;A_{2}=\mu \left(\alpha +\omega +\mu \right).$$

We obtain

$$\Delta_{1}=m_{0}=A_{1}>0;$$

$${∆}_{2}=\left|\begin{array}{cc} m_{0} & 1\\ 0 & m_{1} \end{array}\right|=m_{0}m_{1}=A_{1}{\cdot A}_{2}>0.$$

This means that $\phi_{4},\phi_{5}$ have negative real parts. The other two eigenvalues $\phi_{6},\phi_{7}$ are the zeros of the function

$$f_{2}\left(\phi \right)=\phi^{2}+A_{3}\phi +A_{4},$$

Here

$${\;A}_{3}=-\beta S_{0}+\lambda_{1}\eta_{1}+\lambda_{2}\eta_{2}+\lambda_{3}\eta_{3}+\mu {+\gamma }_{2}+\mu ,$$

$$\;A_{4}=-\beta S_{0}\gamma_{2}+\lambda_{1}\eta_{1}\gamma_{2}+\lambda_{2}\eta_{2}\gamma_{2}+\lambda_{3}\eta_{3}\gamma_{2}+\mu \gamma_{2}-\beta S_{0}\mu +\lambda_{1}\eta_{1}\mu +\lambda_{2}\eta_{2}\mu +\lambda_{3}\eta_{3}\;\mu +\mu^{2}-\beta S_{0}\lambda_{3}\eta_{3}.$$

When $R_{c}<1$, $A_{3}>0,A_{4}>0$, according to the Routh-Hurwitz discriminant, the characteristic roots $\phi_{6},\;\phi_{7}$ have negative real parts.

When $R_{c}>1$, $A_{4}<0$, $f\left(\phi \right)$ have at least one characteristic root with a positive real part, the system (1) is unstable at the disease-free equilibrium point $P_{0}$.

In summary, when $R_{c}<1$, all eigenvalues of the Jacobi matrix of system (1) have negative real parts, and the system (1) is locally asymptotically stable at the disease-free equilibrium point $P_{0}$; when $R_{c}>1$, the system (1) is unstable at the disease-free equilibrium point $P_{0}$. □

### 2.4. Endemic Equilibrium Point and Its Stability

When $A^{*}\neq 0$, in the system (1), the following endemic equilibrium point exists:

$$P^{*}=\left(S^{*}{,E}^{*}{,A}^{*},{C,}^{*}K^{*},V^{*},R^{*}\right).$$

Here

$$S^{*}=\frac{\Lambda \left(\omega +\mu \right)\lambda_{1}\eta_{1}\left(\gamma_{2}+\mu \right)}{\mu \lambda_{1}\eta_{1}\left(\gamma_{2}+\mu \right)\left(\alpha -\omega -\mu \right)-\beta \left(\omega +\mu \right)\left(\varepsilon qA^{*}+\left(1-q\right)\rho A^{*}+\mu A^{*}\right)\left(\gamma_{2}+\mu +\lambda_{3}\eta_{3}\right)},$$

$$E^{*}=\frac{\varepsilon qA^{*}+\left(1-q\right)\rho A^{*}+\mu A^{*}}{\lambda_{1}\eta_{1}},$$

$$C^{*}=\frac{\lambda_{2}\eta_{2}\left(\varepsilon qA^{*}+\left(1-q\right)\rho A^{*}+\mu A^{*}\right)+\lambda_{1}\eta_{1}q\varepsilon A^{*}}{\lambda_{1}\eta_{1}\left(\gamma_{1}+\mu \right)},$$

$$K^{*}=\frac{\lambda_{3}\eta_{3}\left(\varepsilon qA^{*}+\left(1-q\right)\rho A^{*}+\mu A^{*}\right)}{\lambda_{1}\eta_{1}\left(\gamma_{2}+\mu \right)},$$

$$V^{*}=\frac{\Lambda \alpha {\lambda_{1}\eta }_{1}\left(\gamma_{2}+\mu \right)}{\mu \lambda_{1}\eta_{1}\left(\gamma_{2}+\mu \right)\left(\alpha -\omega -\mu \right)-\beta \left(\omega +\mu \right)\left(\varepsilon qA^{*}+\left(1-q\right)\rho A^{*}+\mu A^{*}\right)\left(\gamma_{2}+\mu +\lambda_{3}\eta_{3}\right)},$$

$$R^{*}=\frac{(1-q)\rho A^{*}}{\mu }.$$

**Theorem** **2.**  *When* $R_{c}>1$ *the system (1) has a unique endemic equilibrium point*.

**Proof.**  Using the equilibrium equation of the system (1), we can obtain

$$K^{*}=\frac{\lambda_{3}\eta_{3}E^{*}}{\gamma_{2}+\mu },$$

$$A^{*}=\frac{\lambda_{1}\eta_{1}E^{*}}{q\varepsilon +\left(1-q\right)\rho +\mu },$$

$$C^{*}=\frac{\lambda_{2}\eta_{2}E^{*}}{\gamma_{1}+\mu }+\frac{{q\varepsilon \lambda }_{1}\eta_{1}E^{*}}{\left(\gamma_{1}+\mu \right)\left(q\varepsilon +\left(1-q\right)\rho +\mu \right)},$$

$$R^{*}=\frac{\left(1-q\right)\rho \lambda_{1}\eta_{1}E^{*}}{\left(q\varepsilon +\left(1-q\right)\rho +\mu \right)\mu },$$

$$S^{*}=\frac{\Lambda }{\beta \left(1+\frac{\lambda_{3}\eta_{3}}{\gamma_{2}+\mu }\right)E^{*}+\frac{\alpha \mu }{\omega +\mu }+\mu },$$

$$V^{*}=\frac{\alpha }{\omega +\mu }\cdot \frac{\Lambda }{\beta \left(1+\frac{\lambda_{3}\eta_{3}}{\gamma_{2}+\mu }\right)E^{*}+\frac{\alpha \mu }{\omega +\mu }+\mu },$$

Furthermore, according to the equilibrium equation we obtain

$$\Lambda -\mu S^{*}-\lambda_{1}\eta_{1}E^{*}-\lambda_{2}\eta_{2}E^{*}-\lambda_{3}\eta_{3}E^{*}-\mu E^{*}+\mu V^{*}=0.$$

From this, we can obtain

(2) $$E^{*}=\frac{\Lambda \beta \left(1+\frac{\lambda_{3}\eta_{3}}{\gamma_{2}+\mu }\right)-\left(\frac{\alpha \mu }{\omega +\mu }+\mu \right)\left(\lambda_{1}\eta_{1}+\lambda_{2}\eta_{2}+\lambda_{3}\eta_{3}+\mu \right)}{-\beta \left(1+\frac{\lambda_{3}\eta_{3}}{\gamma_{2}+\mu }\right)\left(\lambda_{1}\eta_{1}+\lambda_{2}\eta_{2}+\lambda_{3}\eta_{3}+\mu \right)}.$$

When

(3) $$R_{c}>1,\beta S_{0}\left(\gamma_{2}+\mu +\lambda_{3}\eta_{3}\right)>\left(\gamma_{2}+\mu \right)\left(\lambda_{1}\eta_{1}+\lambda_{2}\eta_{2}+\lambda_{3}\eta_{3}+\mu \right).$$

Put

$$S_{0}=\frac{\Lambda }{\mu \left(\frac{\alpha }{\omega +\mu }+1\right)}.$$

into Equation (3) and simplify so that we obtain

$$\beta \Lambda \left(1+\frac{\lambda_{3}\eta_{3}}{\gamma_{2}+\mu }\right)>\mu \left(\lambda_{1}\eta_{1}+\lambda_{2}\eta_{2}+\lambda_{3}\eta_{3}+\mu \right)\left(\frac{\alpha }{\omega +\mu }+1\right)$$

$$1-R_{c}=\frac{\mu \left(\frac{\alpha }{\omega +\mu }+1\right)\left(\lambda_{1}\eta_{1}+\lambda_{2}\eta_{2}+\lambda_{3}\eta_{3}+\mu \right)-\beta \Lambda \left(1+\frac{\lambda_{3}\eta_{3}}{\gamma_{2}+\mu }\right)}{\mu \left(\frac{\alpha }{\omega +\mu }+1\right)\left(\lambda_{1}\eta_{1}+\lambda_{2}\eta_{2}+\lambda_{3}\eta_{3}+\mu \right)}.$$

Here

$$E^{*}=\frac{\mu \left(\gamma_{2}+\mu \right)\left(\alpha +\omega +\mu \right)\left(R_{c}-1\right)}{\beta \left(\gamma_{2}+\mu +\lambda_{3}\eta_{3}\right)\left(\omega +\mu \right)}.$$

when $R_{c}>1,E^{*}>0.$

Therefore, when $R_{c}>1$*,* the system (1) has a unique endemic equilibrium point $P^{*}.$ □

**Theorem** **3.**  *When* $R_{c}>1,$ *the system (1) is locally asymptotically stable at the endemic equilibrium point* $P^{*}$.

**Proof.**  Let the Jacobi matrix of the system (1) at the endemic equilibrium point $P^{*}$ be J.

$$\left[\begin{array}{ccc} -\alpha -\mu -\beta \left(E^{*}+K^{*}\right) & -\beta S^{*} & 0\\ \beta \left(E^{*}+K^{*}\right) & \beta S^{*}-\lambda_{1}\eta_{1}-\lambda_{2}\eta_{2}-\lambda_{3}\eta_{3}-\mu \; & 0\\ \begin{array}{c} 0\\ 0\\ \begin{array}{c} \begin{array}{c} 0\\ 0 \end{array}\\ \alpha \end{array} \end{array} & \begin{array}{c} \lambda_{1}\eta_{1}\\ \lambda_{2}\eta_{2}\\ \begin{array}{c} \begin{array}{c} \lambda_{3}\eta_{3}\\ 0 \end{array}\\ 0 \end{array} \end{array} & \begin{array}{c} -q\varepsilon -\left(1-q\right)\rho -\mu \\ q\varepsilon \\ \begin{array}{c} 0\\ \begin{array}{c} \left(1-q\right)\rho \\ 0 \end{array} \end{array} \end{array} \end{array}\;\;\;\;\;\begin{array}{ccc} 0 & \;\;\begin{array}{cc} -\beta S^{*} & \;\;\;0 \end{array} & \omega \\ 0 & \;\;\;\;\;\begin{array}{cc} \beta S^{*}\;\; & \;\;0 \end{array} & 0\\ \begin{array}{c} 0\\ -\gamma_{1}-\mu \\ \begin{array}{c} \begin{array}{c} 0\\ 0 \end{array}\\ 0 \end{array} \end{array} & \begin{array}{c} \;\;\;\;\;\begin{array}{cc} \;0\;\;\;\; & \;\;\;0 \end{array}\\ \;\;\;\;\;\;\begin{array}{cc} 0 & \;\;\;\;\;\;\;0 \end{array}\\ \begin{array}{c} \begin{array}{cc} -\gamma_{2}-\mu & 0 \end{array}\\ \;\;\;\;\;\;\begin{array}{c} \begin{array}{cc} 0 & \;\;\;\;-\mu \end{array}\\ \begin{array}{cc} 0 & \;\;\;\;\;\;\;\;0 \end{array} \end{array} \end{array} \end{array} & \begin{array}{c} 0\\ 0\\ \begin{array}{c} \begin{array}{c} 0\\ 0 \end{array}\\ -\omega -\mu \end{array} \end{array} \end{array}\right].$$

The characteristic polynomial is obtained by calculating

$$f\left(\phi \right)=g_{1}\left(\phi \right)\cdot g_{2}\left(\phi \right),$$

where

$$g_{1}\left(\phi \right)=\left(\phi +\mu \right)\left(\phi +\gamma_{1}+\mu \right)\left(\phi +q\varepsilon +\left(1-q\right)\rho +\mu \right),$$

$$g_{2}\left(\phi \right)=\left(\phi +\omega +\mu \right)\{\left(\phi +\gamma_{2}+\mu \right)[\left(\phi +\alpha +\mu \right)\left(\phi -\beta S^{*}+\lambda_{1}\eta_{1}+\lambda_{2}\eta_{2}+\lambda_{3}\eta_{3}+\mu \right)+\;\beta S^{*}\left(E^{*}+K^{*}\right)\left(\phi +\lambda_{1}\eta_{1}+\lambda_{2}\eta_{2}+\lambda_{3}\eta_{3}+\mu \right)]-\beta S^{*}\lambda_{3}\eta_{3}\left(\phi +\alpha +\mu \right)\}$$

$$-\alpha \omega \left[\left(\phi -\beta S^{*}+\lambda_{1}\eta_{1}+\lambda_{2}\eta_{2}+\lambda_{3}\eta_{3}+\mu \right)\left(\phi +\gamma_{2}+\mu \right)-\beta S^{*}\lambda_{3}\eta_{3}\right].$$

It is easy to know that all the eigenvalues of $g_{1}\left(\phi \right)$ have negative real parts, and the other four eigenvalues are the zeros of the function $g_{2}\left(\phi \right)$.

From $g_{2}\left(\phi \right)=0$ we obtain

$$\beta S^{*}\lambda_{3}\eta_{3}\left(\phi +\mu \right)\left(\phi +\alpha +\omega +\mu \right)=\left(\phi +\mu \right)\left(\phi +\alpha +\omega +\mu \right)\left(\phi +\gamma_{2}+\mu \right)\left(\phi -\beta S^{*}+\lambda_{1}\eta_{1}+\lambda_{2}\eta_{2}+\lambda_{3}\eta_{3}+\mu \right)\;+\beta S^{*}\left(E^{*}+K^{*}\right)\left(\phi +\omega +\mu \right)\left(\phi +\gamma_{2}+\mu \right)\left(\phi +\lambda_{1}\eta_{1}+\lambda_{2}\eta_{2}+\lambda_{3}\eta_{3}+\mu \right).$$

Here

(4) $$1=\frac{\left(\phi +\gamma_{2}+\mu \right)\left(\phi -\beta S^{*}+\lambda_{1}\eta_{1}+\lambda_{2}\eta_{2}+\lambda_{3}\eta_{3}+\mu \right)}{\beta S^{*}\lambda_{3}\eta_{3}}\;+\frac{\beta S^{*}\left(E^{*}+K^{*}\right)\left(\phi +\omega +\mu \right)\left(\phi +\gamma_{2}+\mu \right)\left(\phi +\lambda_{1}\eta_{1}+\lambda_{2}\eta_{2}+\lambda_{3}\eta_{3}+\mu \right)}{\beta S^{*}\lambda_{3}\eta_{3}\left(\phi +\mu \right)\left(\phi +\alpha +\omega +\mu \right)}.$$

Assume that $Re\left(\phi \right)\geq 0$ when $g_{2}\left(\phi \right)=0$. Taking the norm on the right side of the equal sign of Equation (4) can obtain

(5) $$\left|\frac{\left(\phi +\gamma_{2}+\mu \right)\left(\phi -\beta S^{*}+\lambda_{1}\eta_{1}+\lambda_{2}\eta_{2}+\lambda_{3}\eta_{3}+\mu \right)}{\beta S^{*}\lambda_{3}\eta_{3}}+\frac{\beta S^{*}\left(E^{*}+K^{*}\right)\left(\phi +\omega +\mu \right)\left(\phi +\gamma_{2}+\mu \right)\left(\phi +\lambda_{1}\eta_{1}+\lambda_{2}\eta_{2}+\lambda_{3}\eta_{3}+\mu \right)}{\beta S^{*}\lambda_{3}\eta_{3}\left(\phi +\mu \right)\left(\phi +\alpha +\omega +\mu \right)}\right|\;>\frac{\left(\gamma_{2}+\mu \right)\left(-\beta S^{*}+\lambda_{1}\eta_{1}+\lambda_{2}\eta_{2}+\lambda_{3}\eta_{3}+\mu \right)}{\beta S^{*}\lambda_{3}\eta_{3}}=1.$$

We can find that the results of Equations (4) and (5) contradict each other, so the roots of $g_{2}\left(\phi \right)$ also have negative real parts.

When $R_{c}>1$, all the characteristic roots of the Jacobi matrix of system (1) have negative real parts, so the system (1) is locally asymptotically stable at the endemic equilibrium point $P^{*}$. □

## 3. Results

### 3.1. Parameter Values and Sources

If the average incubation period of the hepatitis B virus infection is 105 days [6], we obtain $\eta_{1}=\eta_{2}=\eta_{3}=3.4$. Because the GAVI project entails vaccinating all newborns [21], the vaccination rate α=1 is determined and fitted. The parameters $\lambda_{1},\lambda_{2}$ are obtained by data fitting, and $\lambda_{1}+\lambda_{2}+\lambda_{3}=1$. $\lambda_{3}$ is obtained through calculation. $\gamma_{1},\gamma_{2}$ are obtained from the mortality rate of hepatitis B in China published by the Chinese Center for Disease Control and Prevention [6], and $\gamma_{1}{=\gamma }_{2}=0.0461.$

The initial value of S is taken from the total national population in 2003 published by the National Bureau of Statistics of China [22]. The initial value of C is the total number of hepatitis B cases nationwide in 2003 published by the National Health Commission of China [3]. The initial value of A is taken by the ratio of the number of acute hepatitis B cases to chronic hepatitis B cases of 1:5. The initial value of V is taken from the total number of people vaccinated against hepatitis B nationwide in 2002, published in [22], and the initial value of R is calculated by known parameters. So, in this case, S(0) = 1,292,270,000, E(0) = 250,000, A(0) = 143,802, C(0) = 719,011, K(0) = 180,000, V(0) = 6,540,000, R(0) = 68,737.

### 3.2. Fitting Result

**Figure 3:**
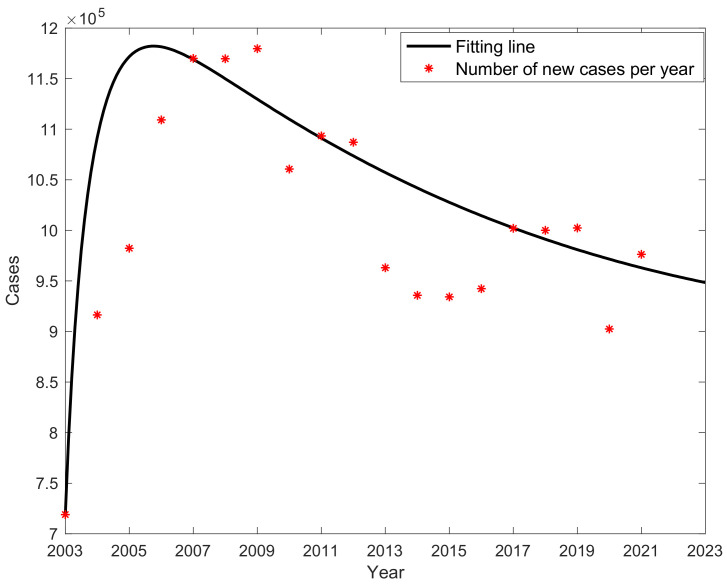
Fitted graph of the number of hepatitis B cases in China from 2003 to 2021.

**Figure 4:**
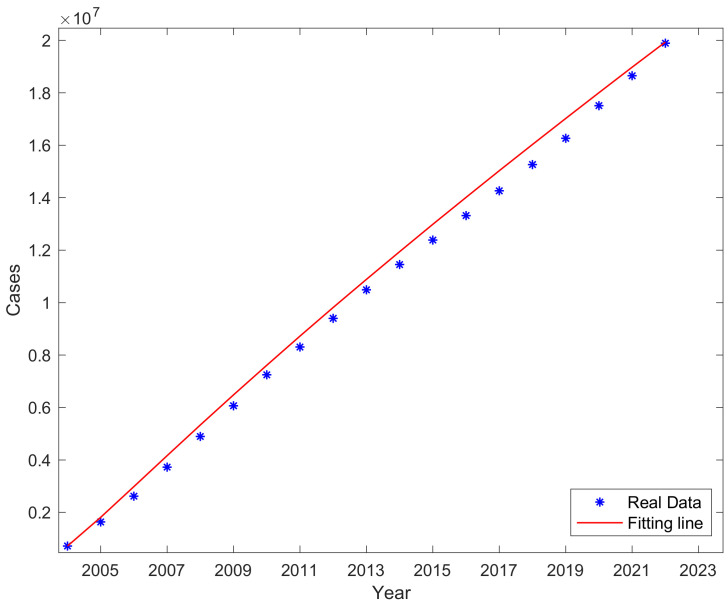
Real cumulative cases and fitted cumulative cases.

For each year’s new case data, a nonlinear least-squares fitting was conducted, resulting in values for parameters β,$\;\lambda_{1},\lambda_{2}$; the results are shown in Table 1, and it can be seen that the proportion of potentially infectious cases in the compartment is as high as 30.49%. Using the Runge–Kutta methods to solve differential equations and programming in MATLAB for fitting, the results of the fitting are shown in Figure 3. The red marks represent the actual data of newly reported cases each year, and the black curve represents the fitted results obtained through programming. After observing, it was found that the development trend of the fitted curve closely aligned with the actual incidence trend, and after reaching its peak in 2006, it consistently followed a descending trend. The curve of actual cumulative cases in comparison to the fitted cumulative case curve are shown in Figure 4; the fitting demonstrated a good performance with the trend of the fitted curve aligning well with the actual curve.

### 3.3. Sensitivity Analysis

**Figure 5:**
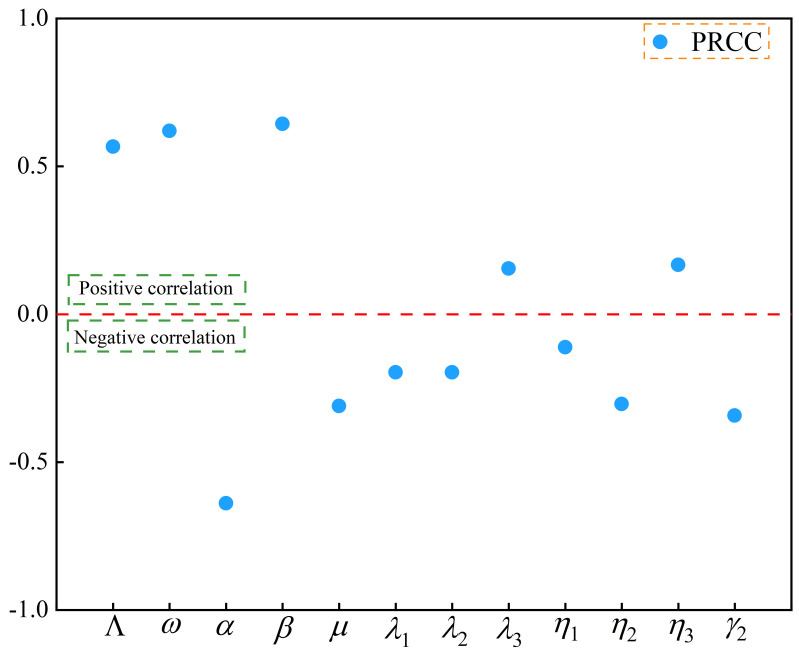
Correlation between the control reproduction number and the parameters of the model.

A way to find the most sensitive epidemiological parameters, which should be prioritized when controlling infectious diseases, can be done by determining the strength of the correlations between each parameter in the model and the control reproduction number $R_{c}$ [23]. We conducted a sensitivity analysis of control reproduction numbers using the partial order correlation coefficient (PRCC) [24] in terms of the magnitude of the absolute value of the correlation coefficient. The results of the sensitivity analysis are presented in Figure 5 and Table 2. Figure 5 provides the correlation between the number of control reproduction numbers and each parameter of the model in the form of an interval plot, where the parameter located above the red dashed line indicates a positive correlation with $R_{c}$, and below it indicates a negative correlation with $R_{c}$. Table 2 contains the specific values of the correlation coefficients of the control reproduction number with respect to each parameter of the model. The control reproduction number, the number of new births rate every year Λ, the transmission rate β, the vaccine failure rate ω, the transfer rate from E to K $\eta_{3}$, and the conversion rate of E to K $\lambda_{3}$ are the most sensitive parameters. At the same time, improving the effectiveness of the hepatitis B vaccine and controlling the transmission of the hepatitis B virus from patients can significantly reduce the number of hepatitis B cases.

### 3.4. The Number of A, C, and Potential Virus Infections K with Time

**Figure 6:**
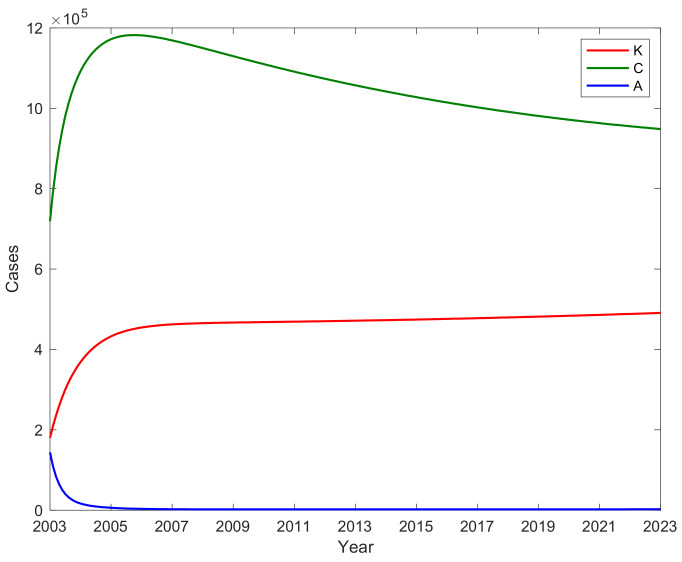
The number of acute infection individuals A, chronic HBV infections C, and potential hepatitis B virus infections K with time.

We substituted the fitted parameters into the control regeneration number formula and calculated the control reproduction number $R_{c}$ = 1.741. This control reproduction number served as a critical indicator in assessing the potential for disease transmission control in the context of hepatitis B. Furthermore, the application of the fitted parameters allowed for the calculation of various disease-related quantities, which are illustrated in Figure 6. This figure presents trends in the number of acute hepatitis B infections (A), chronic hepatitis B infections (C), and potentially infectious hepatitis B virus infections (K). The number of chronic hepatitis B infections C peaked in 2006 with 1,181,405 individuals affected at that time and subsequently experienced a gradual decline. The number of acute hepatitis B infections A initially decreased to 2471 in 2006, stabilizing at around 2188 after 2006. Based on the fitted data, the average number of potentially infectious hepatitis B virus infections was estimated to be 449,535 (95% CI [415,651, 483,420]); this estimate reflects the underlying reservoir of potentially infectious hepatitis B virus infections that could contribute to ongoing transmission dynamics. Remarkably, the number of potentially infectious hepatitis B virus infections K has been consistently increasing since 2003, reaching 466,907 in 2009 and stabilizing at 466,907 after 2009. This suggests that the GAVI project, which was implemented from 2002 to 2007 and only provided booster immunization to children under 15 years of age, had a minimal impact on the number of potentially infectious infections among adults.

However, in a promising development, the number of new cases of acute infections in the subsequent eight-year period exerted a significant control effect. This effect was manifested in a noteworthy reduction in the incidence of infections among adolescents transitioning into adulthood. This implies that while the GAVI project may not have substantially influenced adult infections, its impact is more pronounced in preventing new infections among those entering adulthood.

In summation, the utilization of fitted parameters has facilitated a deeper understanding of the disease dynamics surrounding hepatitis B. The calculations of control reproduction numbers and various infection categories offer valuable insights into the effectiveness of intervention strategies, such as vaccination campaigns and their distinct impacts on different age groups within the population. These findings underscore the nuanced nature of disease control efforts and emphasize the importance of tailoring interventions to specific demographic segments.

### 3.5. The Impact of Parameter $\lambda_{3}$ on Hepatitis B Virus Transmission

**Figure 7:**
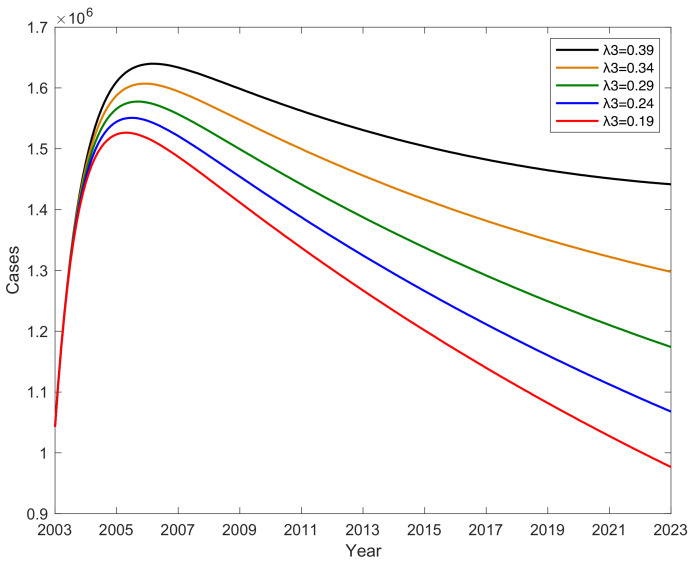
The impact of parameter $\lambda_{3}$ on hepatitis B virus transmission.

The conversion rate $\lambda_{3}$ from latently infected E to potential hepatitis B virus carrier K has an impact on the number of hepatitis B cases; this impact becomes particularly evident when considering the interplay between different conversion rates within the disease transmission model. When the conversion rate $\lambda_{2}$ from latently infected E to chronic hepatitis B-infected C increases, the conversion rate $\lambda_{1}$ from latently infected E to acute hepatitis B patient A remains constant, and the conversion rate $\lambda_{3}$ from latently infected E to potential hepatitis B virus carrier K gradually decreases, as shown in Figure 7. This indicates that the number of new cases of hepatitis B per year decreases as $\lambda_{3}$ decreases. This trend is consistent with the hypothesis that fewer individuals transition from latent infection to potential carrier status, resulting in a reduction in new cases of hepatitis B. For instance, if $\lambda_{3}$ decreases by 0.2, the average annual count of new hepatitis B cases decreases by an average of 257,882. This observation highlights the sensitivity of disease dynamics to changes in conversion rates, particularly those that influence progression to potential carrier status. Therefore, increasing the level of hepatitis B testing in the population will reduce the proportion of potential hepatitis B virus infections. By analyzing the conversion rate $\lambda_{3}$ from latently infected E to potential hepatitis B virus carrier K, we uncovered the mechanisms influencing the number of hepatitis B cases. Research findings indicate that a gradual reduction in the conversion rate $\lambda_{3}$ from latently infected E to potential hepatitis B virus carrier K will significantly decrease the annual incidence of new cases. This discovery underscores the significance of intervention measures targeting potential hepatitis B carriers in controlling the spread of the hepatitis B virus.

### 3.6. Impact of Vaccine Failure Rate ω on Potential Hepatitis B Virus Infections 

**Figure 8:**
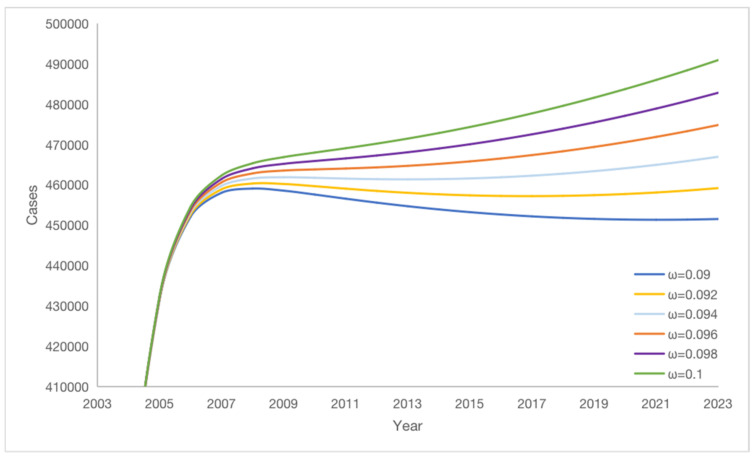
The effect of vaccine failure rate ω on potential virus infections K.

Simulation results indicate that the vaccine failure rate ω affects the change in the number of potential hepatitis B virus infections K. As the vaccine failure rate ω decreases, the number of potential hepatitis B virus infections K gradually decreases, but K decreases more and more slowly (see Figure 8). Lower vaccine failure rates correspond to fewer potential hepatitis B virus infections. A compelling observation emerges from these findings: lower vaccine failure rates indeed correspond to fewer potential hepatitis B virus infections. Reducing the vaccine failure rate ω by 1% results in the average number of potential hepatitis B virus infections K decreasing by 17,454. This underscores the substantial impact that the mitigation of the vaccine failure rate can have in curbing the prevalence of potential carriers. Therefore, mitigating the number of potential hepatitis B virus infections K can be achieved by decreasing the vaccine failure rate. The simulation results reveal that reducing vaccine failure rate ω can significantly decrease the number of potential hepatitis B virus carriers (K). However, as the vaccine failure rate ω decreases, the rate of carrier reduction gradually slows, which might be associated with ongoing infections in a portion of the population. Thus, lowering the vaccine failure rate ω remains an effective strategy to reduce the number of carriers, but the impact of ongoing infections also needs to be taken into consideration. In conclusion, the simulation results underscore the multifaceted nature of disease control efforts. They highlight the potential of reducing the vaccine failure rate to significantly mitigate the prevalence of potential carriers. However, the presence of ongoing infections necessitates a holistic strategy that combines vaccine efficacy improvements with measures addressing the ongoing transmission dynamics. Such a comprehensive approach holds the key to achieving substantial and sustained reductions in the number of hepatitis B virus carriers.

The vaccine failure rate ω affects the proportion of potential hepatitis B virus infections K, as shown in Table 3. This table portrays the intricate relationship between the vaccine failure rate and the extent of potential infections within the population. If the vaccine failure rate ω is reduced by 2%, the proportion of potential hepatitis B virus infections K decreases by 1.41% on average. This outcome signifies a direct and proportionate relationship between changes in the vaccine failure rate and the prevalence of potential infections. 

As the vaccine failure rate changes, the ratio of the number of potential hepatitis B virus infections K varies in response to the proportion of K in the sick person’s compartments (A + C + K), as shown in Table 3. When the vaccine failure rate ω is lower, the proportion of K within the compartment of infected individuals is smaller, resulting in fewer potential hepatitis B virus infections K. Therefore, reducing the vaccine failure rate can effectively reduce the number of potential hepatitis B virus infections K. 

Collectively, these insights accentuate the pivotal role that the vaccine failure rate plays in shaping the landscape of potential hepatitis B virus infections. The findings highlight the need for continuous efforts to improve vaccine efficacy and minimize failure rates. Such endeavors hold the promise of not only reducing the burden of potential infections but also contributing to more effective disease control strategies. 

### 3.7. Effect of Vaccine Failure Rates ω on the Number of C When Vaccination Rate Is 100%

**Figure 9:**
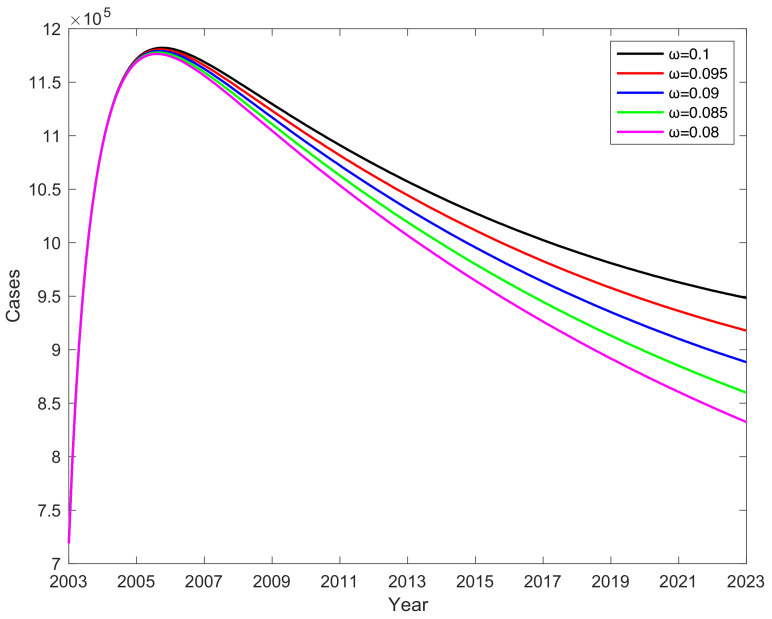
Effect of vaccine failure rate ω on the number of chronic HBV infections.

In China, vaccine coverage for children under five years old has exceeded 95% and almost 100% since 2014. When the vaccination rate is 100% (α = 1), the effect of different vaccine failure rates ω on the number of chronic hepatitis B infections is examined. The rate of change in the number of chronic hepatitis B infections is determined by the vaccine failure rate ω, and the number of chronic hepatitis B infections decreases as the vaccine failure rate ω decreases, as shown in Figure 9. A reduction in the vaccine failure rate ω by 2% results in an average decrease of 51,993 in the number of chronic hepatitis B cases. This indicates that lower vaccine failure rates correspond to fewer cases of chronic hepatitis B. Lowering the vaccine failure rate ω can significantly decrease the number of chronic hepatitis B cases, further confirming the importance of vaccines in preventing hepatitis B transmission. Governments should continue to enhance the oversight of vaccine quality to ensure both their effectiveness and safety. By maintaining high levels of vaccine coverage and reducing vaccine failure rates, countries can enhance their ability to prevent and control the spread of infectious diseases such as hepatitis B, thus safeguarding public health more effectively.

### 3.8. Effect of Vaccination Rates α on the Number of C When Vaccine Failure Rate Is 0.1

**Figure 10:**
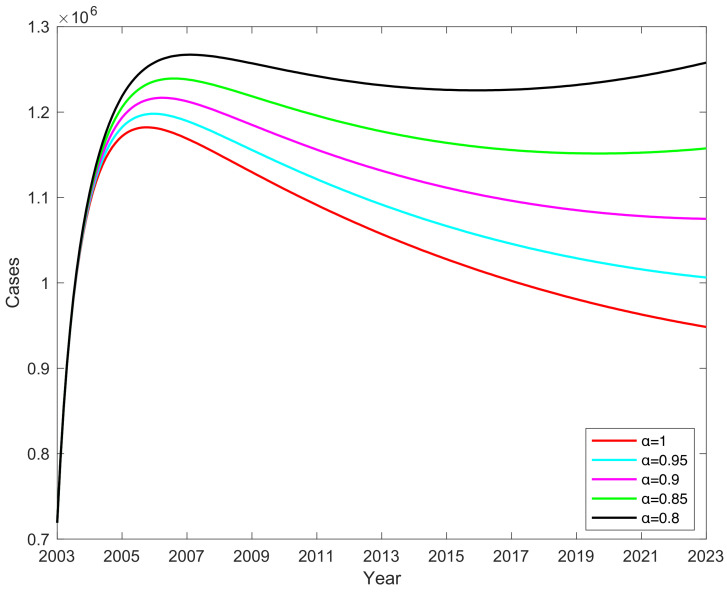
Effect of vaccination rate α on the number of chronic HBV infections C.

When the vaccine failure rate ω is 0.1, different vaccination rates α will have an impact on the number of chronic hepatitis B infections, shown in Figure 10. When the vaccine failure rate ω = 0.1, from which it can be seen that the vaccination rate α determines the rate of change in the number of chronic hepatitis B infections, the number of chronic HBV infections decreases as the vaccination rate α increases, increasing α by 20%; the number of chronic hepatitis B infections decreases by 169,469 on average, indicating that the higher the vaccination rate, the lower the number of chronic hepatitis B cases. It shows that as the vaccine coverage becomes smaller, the peak of chronic hepatitis B infections increases and occurs later than when α = 1. The research findings reveal a close relationship between vaccination rates α and the incidence of chronic hepatitis B cases, which holds significant implications for formulating hepatitis B virus prevention and control strategies. However, it is important to acknowledge that the increase in vaccination rates α is influenced not only by individual choices but also by various factors including social awareness campaigns, policy support, and the allocation of medical resources. Therefore, when formulating and implementing vaccination plans, it is crucial to comprehensively consider these factors in order to achieve higher vaccination rates.

### 3.9. Effect on $R_{c}$

$R_{c}$ is the threshold value to determine whether the disease is extinct or not. When $R_{c}<1$, the disease gradually dies out; when $R_{c}>1$, the disease develops into an endemic disease [7].

**Figure 11:**
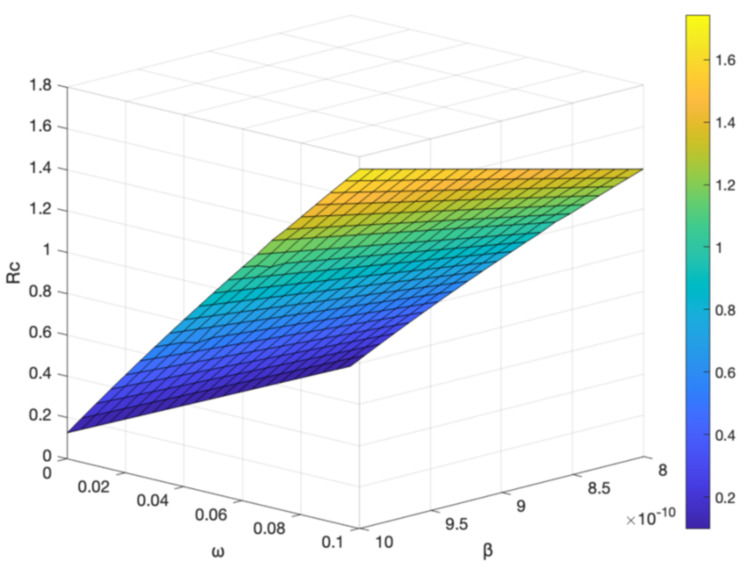
Effect of the vaccine failure rate ω and the basal transmission rate β on $R_{c}$.

**Figure 12:**
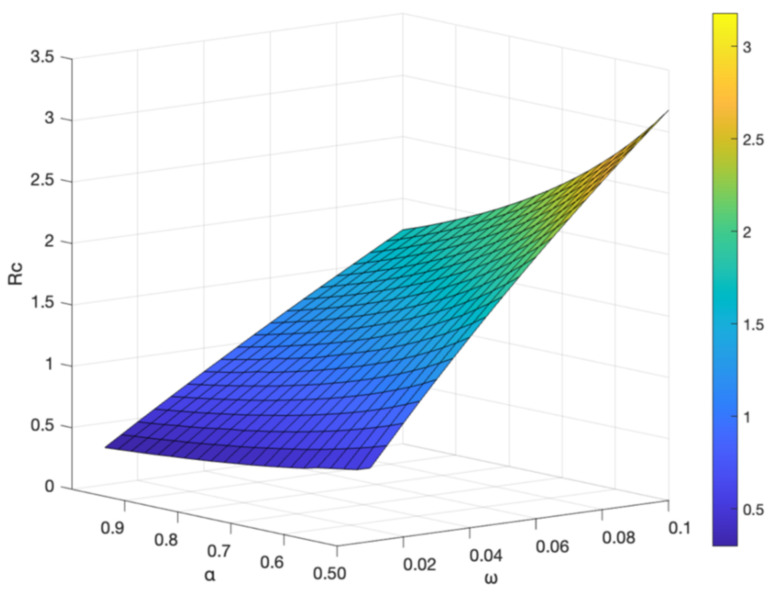
Effect of the vaccine failure rate ω and the vaccination rate α on $R_{c}$.

The interplay between vaccine-related factors and disease transmission dynamics is a critical aspect of understanding and effectively managing infectious diseases, such as hepatitis B. In this context, the vaccine failure rate (ω), the basal transmission rate (β), and the vaccination rate (α) play pivotal roles in shaping the control reproduction number ($R_{c}$), a fundamental parameter that indicates the potential for the spread of disease within a population. By analyzing Figure 11 and Figure 12, we can gain valuable insights into how these factors influence $R_{c}$ and subsequently devise more effective disease control strategies.

Figure 11 illustrates the relationship between $R_{c}$ and both the vaccine failure rate (ω) and the basal transmission rate (β). As observed, an increase the value either ω or β leads to the increase the value $R_{c}$. This outcome signifies that higher vaccine failure rates and elevated transmission rates contribute to greater disease propagation potential within the population. In practical terms, a higher $R_{c}$ implies a larger number of secondary infections arising from each primary infection, which could lead to more widespread disease outbreaks.

Figure 12 delves into the impact of the vaccination rate (α) and the vaccine failure rate (ω) on $R_{c}$. It is noteworthy that $R_{c}$ decreases as vaccination coverage (α) increases and vaccine failure rate (ω) decreases. This suggests that higher vaccination coverage and lower vaccine failure rate can effectively reduce the likelihood of disease transmission. In other words, higher vaccination coverage combined with lower vaccine failure rate leads to a decrease in $R_{c}$, which suggests that hepatitis B spreads more slowly in the population.

From these observations, it becomes evident that enhancing hepatitis B vaccine coverage is a pivotal strategy for controlling the spread of the virus. While focusing on vaccinating newborns is essential, the findings strongly advocate for the reinforcement of vaccination efforts across diverse age groups. In doing so, population-wide immunity levels can be raised, thereby reducing the overall transmission potential of hepatitis B. In summary, the intricate interplay between vaccine failure rates, basal transmission rates, and vaccination rates has a profound impact on the control reproduction number ($R_{c}$) and, consequently, on the dynamics of hepatitis B transmission. The insights gleaned from Figure 11 and Figure 12 underscore the significance of comprehensive vaccination strategies to curtail the spread of the disease effectively. 

## 4. Conclusions

The main focus of this paper is to estimate the number of potential hepatitis B virus carriers. No research has been conducted on this aspect, which distinguishes this paper from others. Through fitting, the quantity of potential hepatitis B virus carriers along with influencing factors can be determined. Additionally, this study also examines vaccine failure rates and vaccination rates. Research on vaccine failure rates can be found in other literature, and from these sources, the following conclusions have been drawn: Haile Habenom et al. found that, through vaccination coverage in the population, it is not difficult to conclude that implementation of all control strategies (i.e., vaccination and having less contact rates using isolation strategy) can help us to reduce infectious compartments and control the spread of the hepatitis B virus [15]; Ramses Djidjou Demasse et al. studied the impact of vaccination and found that vaccination of young adults can greatly reduce the total number of infected individuals and is crucial to apply during the first few months of disease detection [16]; Williams JR et al. illustrated the temporal impact of different mass vaccination options on the prevalence of heterosexual and homosexual carriers and ensured that the mass vaccination of infants was effective in reducing the number of hepatitis B virus infections [25]. In our paper, it can be observed that with the increase in vaccination rates, the number of potential hepatitis B virus carriers continues to decrease. Therefore, we should enhance vaccination efforts among susceptible populations to minimize the number of afflicted individuals.

China is one of the countries with the highest rates of hepatitis B in the world with the incidences of hepatitis B rising continuously. Accurately estimating the number of potential hepatitis B virus carriers is crucial for the prevention and control of hepatitis B. Therefore, in this paper, a dynamic model of hepatitis B virus transmission dynamics with vaccination was developed based on the transmission mechanism of the hepatitis B virus to study the number of potential hepatitis B virus carriers. By utilizing nonlinear least-squares fitting, this study estimates that the potential hepatitis B virus carriers K are 449,535 (95%CI [415,651, 483,420]), constituting 30.49% of hepatitis B patients; this underscores the significant reservoir of latent infections that could potentially contribute to future transmission cycles. The control reproduction number $R_{c}$ = 1.741 for hepatitis B virus transmission in China was obtained according to the fitted parameters and the next generation matrix method; this indicates that the transmission of the hepatitis B virus in China is still in a critical situation, the number of new hepatitis B cases continues to increase at a high level in the population, and additional control is needed to slow the spread of the virus. In conclusion, this study’s comprehensive analysis provides a nuanced understanding of the dynamics of hepatitis B virus transmission within China. By integrating a dynamic mathematical model with empirical data and sophisticated analysis techniques, the research offers a concrete estimation of potential virus carriers and a vital assessment of the transmission situation. These insights form a solid foundation for informed decision making in public health policies and underscore the urgency of intensified efforts to contain the spread of the hepatitis B virus within China’s population.

This study examined the effect of different parameters on hepatitis B virus transmission. And it was shown that the conversion rate $\lambda_{3}$ from exposed E to potential hepatitis B virus carrier K affects the transmission of the hepatitis B virus. When the vaccination rate α increases and the vaccine failure rate ω decreases, the control reproduction number $R_{c}$ decreases. This analysis revealed that reducing the vaccine failure rate, increasing the vaccination rate, and increasing hepatitis B testing among the public are essential for reducing the number of hepatitis B cases and controlling the number of potential hepatitis B virus infections K. Therefore, encouraging hepatitis B vaccination for all types of people and increasing the rate of hepatitis B vaccination can effectively control the spread of the hepatitis B virus.

This result revealed that the number of potential infectious hepatitis B virus infections is high, contributing to the persistent rise in hepatitis B patients in China. To better control the transmission of the hepatitis B virus, one optional prevention and control strategy is to increase vaccination of different age groups. At the same time, it is necessary to increase publicity for the prevention of the transmission of the hepatitis B virus to help the public correctly understand the transmission of hepatitis B, ensure adequate protection, and call on the public to strengthen their own efforts. All these actions will have a positive impact on the prevention and treatment of hepatitis B.

This paper also has some limitations. This study was based only on data of annual hepatitis B incidences. While data on drug users and gender could influence epidemiological evaluations, the official website of the Chinese Health and Welfare Bureau only provided annual hepatitis B incidence figures without drug users or gender data. In our study, we assumed that acute hepatitis B patients and chronic hepatitis B patients no longer exhibit infectiousness; however, it is challenging to substantiate this assumption in real-world scenarios. The latest annual case data indicate significant variations in hepatitis B case numbers across different age groups. In future studies, we intend to explore the impact of age heterogeneity on hepatitis B virus transmission and identify optimal vaccination strategies.

## Figures and Tables

**Table 1:** Value and source of parameter values.

Parameter	Value	Reference	Parameter	Value	Reference
α	1	Estimated	μ	0.007	[22]
ω	0.1	[6]	$\lambda_{1}$	0.11	Fitted
$\eta_{1}$	3.4	Calculated	$\lambda_{2}$	0.5	Fitted
$\eta_{2}$	3.4	Calculated	$\lambda_{3}$	0.39	Calculated
$\eta_{3}$	3.4	Calculated	q	0.8805	[1]
$\gamma_{1}$	0.0461	Calculated	$\gamma_{2}$	0.0461	Calculated
β	${1\times 10}^{-9}$	Fitted	ρ	4	[1]
ε	4	[1]	Λ	16,540,000	[22]

**Table 2:** Specific values of the correlation coefficients of the control reproduction number with respect to each parameter of the model.

Parameter	PRCC
β	0.644
ω	0.620
Λ	0.567
$\eta_{3}$	0.167
$\lambda_{3}$	0.155
$\eta_{1}$	−0.111
$\lambda_{1}$	−0.196
$\lambda_{2}$	−0.196
$\eta_{2}$	−0.303
μ	−0.310
$\gamma_{2}$	−0.342
α	−0.639

**Table 3:** Number of cases of potential hepatitis B virus infections K and the proportion of K in the compartment of diseased individuals.

	ω = 0.08		ω = 0.09		ω = 0.1	
Year	$\frac{K}{A+C+K}$	K	$\frac{K}{A+C+K}$	K	$\frac{K}{A+C+K}$	K
2004	0.28	368,296	0.28	368,431	0.28	368,566
2005	0.30	430,598	0.30	431,571	0.30	432,541
2006	0.29	449,705	0.29	452,167	0.29	454,624
2007	0.28	453,797	0.28	458,081	0.28	462,366
2008	0.28	452,865	0.28	459,110	0.28	465,379
2009	0.28	450,334	0.28	458,592	0.28	466,907
2010	0.30	447,350	0.30	457,632	0.31	468,028
2011	0.29	444,292	0.29	456,603	0.30	469,102
2012	0.29	441,287	0.29	455,625	0.30	470,246
2013	0.31	438,374	0.32	454,738	0.33	471,495
2014	0.32	435,566	0.33	453,952	0.34	472,866
2015	0.32	432,865	0.33	453,272	0.34	474,361
2016	0.31	430,269	0.32	452,698	0.34	475,983
2017	0.30	427,778	0.31	452,229	0.32	477,732
2018	0.30	425,387	0.31	451,865	0.32	479,609
2019	0.30	423,097	0.31	451,603	0.32	481,614
2020	0.32	420,904	0.33	451,444	0.35	483,749
2021	0.30	418,807	0.32	451,386	0.33	486,013

## Data Availability

Available online: http://www.nhc.gov.cn (accessed on 2 June 2023).
